# Nutritional Content Analysis of Crop Residues in Three Agroecologies in East Gojjam Zone

**DOI:** 10.1155/2023/1974081

**Published:** 2023-05-13

**Authors:** Alemu Gashe Desta

**Affiliations:** Debre Markos University, Department of Animal Science, Debre Markos, Ethiopia

## Abstract

Livestock is important and plentiful in Ethiopia, but its potential is not being used to its full extent due to limited supply and inconsistent feed supply. This has a negative impact on livestock productivity in the tropics, where crop residues are used as livestock feed. The analysis of the nutritional content of feed and the measurement of feed production are crucial for maintaining the optimal balance of annual livestock feed and improving livestock production. However, no research has been conducted on the nutritional composition of the crop residues in the East Gojjam zone. Therefore, this study was conducted to analyze the nutritional content of crop residues in the three agroecologies in Ethiopia's East Gojjam zone. Ash, dry matter (DM), organic matter (OM), neutral detergent fiber (NDF), acid detergent fiber (ADF), and acid detergent lignin (ADL) were analyzed in the feed samples. According to the findings, the overall mean content of DM, ash, OM, CP, NDF, ADF, and ADL was 92.3, 5.2, 87.1, 4.1, 71, 56.6, and 9.6%, respectively. The faba bean has the highest CP (6.2%), followed by barley straws (5.5%), vetch haulms (5.5%), *Eragrostis tef* (3.7%), wheat straws (3.2%), and maize stover (2.9%). In general, all crop residues contain CP content below the critical CP level required even for livestock maintenance. The highest NDF was found in barley straw (75.1%), which was followed by wheat straw (72.0%), *Eragrostis tef* straw (70.2%), vetch haulms (69.3%), maize stover (67.4%), and faba bean haulms (67.2%). All types of crop residues were classified as low-quality feed because they contained more than 65% NDF, which could affect feed intake and livestock production. Therefore, crop residues must be improved using mechanical, biological, and chemical methods in order to increase livestock production in the study areas by increasing feed consumption.

## 1. Introduction

Although livestock is very important and abundant in Ethiopia, their potential is not being used to its fullest extent due to limited supply and inconsistent year-round feed supply. This has a negative impact on livestock productivity in the tropics and subtropics, where crop residues are used as livestock feed [1–3]. In Ethiopia, multipurpose ruminants in huge numbers and crop residues in enormous amounts are the norm [4].

Crop residues supply roughly 50% of the total resources of ruminant livestock feed in the mixed cereal-dominated crop and livestock production system in the Ethiopian highlands. Crop residues contributed up to 80% of all feed sources during the year's dry seasons [5]. However, the contribution of crop residues varies according to agroecology, crop variety, and production [5]. Due to the cultivation of grazing pastures to meet the grain requirements of the constantly expanding human population, it is projected that the high dependence on crop residues as livestock feed would only increase [6]. As a result, ruminants mostly consume cereal straws as their stable feed [2, 3, 7].

In the developing tropics and subtropics, crop residues are used as livestock feed [8]. However, crop residues are poor in quality due to high fiber, low protein content [9, 10], poor digestibility [11], low metabolizable energy content, low feed intake, and a low amount of readily available minerals and vitamins [3]. Poor animal nutrition is one of the main factors restricting livestock productivity [8]. The poor quality, nutrient content, and digestibility of crop residues can be improved and degraded using various methods such as grinding, stream processing, chemical methods (such as alkaline treatments and other chemicals, such as sodium hydroxide, sulfur dioxide, urea, or chlorine), and biological methods (such as fungal treatment, inoculants, enzymes, or alternative additives) have been utilized separately or in combination [12–14]. However, improving the nutritional content of crop residues is not very common in Ethiopia due to lack of knowledge, lack of funding, and availability of chemicals for treatment [5, 15].

Analyzing the nutritional content of feed and measuring feed production are crucial for maintaining the optimal balance of annual livestock feed and improving livestock production. The dry matter (DM) yield of crop residues in the East Gojjam zone was investigated only in [16]. However, no research has been conducted on the nutritional composition of the crop residues in the zone. Therefore, this study was conducted to estimate the DM yield of crop residues and analyze the nutritional content of crop residues in the three agroecologies in the East Gojjam zone, Ethiopia.

## 2. Materials and Methods

### 2.1. Description of the Study Area

**Figure 1:**
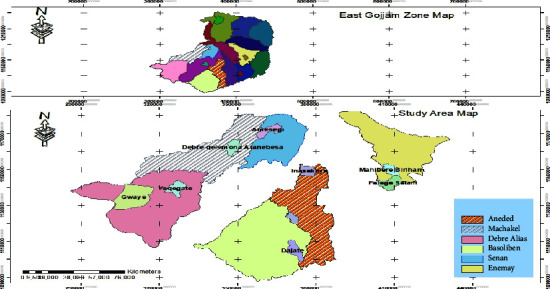
Location map of the six study area districts.

The study was carried out in the East Gojjam zone of Amhara National Regional State, Ethiopia. The zone is found in Ethiopia's Northwestern Highlands between the latitudes of 10° 1′ 46″ and 10° 35′ 12″ N and the longitudes of 37° 23′ 45″ and 37° 55′ 52″ E (Figure 1). It is located 305 and 251 kilometers from Addis Ababa and Bahir Dar, respectively. There are 18 districts in the East Gojjam zone, each with a different elevation and, thus, individual agroecology. The elevation ranges from 1500 to 3577 metres above sea level. The average annual rainfall ranges from 900 to 2000 millimeters, while the average minimum and maximum temperatures are 7 to 15 and 22 to 25 degree Celsius, respectively. According to the East Gojjam Agricultural Office, sheep, goats, and cattle, as well as pack animals (donkeys, horses, and mules), chickens, and bees, were raised in the zone.

**Figure 2:**
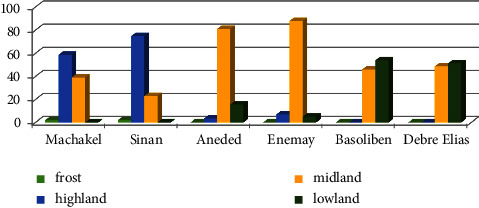
The percentage contribution of agroecology in six sampled study districts.

Figure 2 shows the contribution (%) of agroecology in each sampled district based on information gathered from the East Gojjam Zone Agriculture Office. Machakel district's agroecology is classified as frost, highland (HL), midland (ML), and lowland (LL), with respective values of 2, 59, 39, and 0%, while the agroecology of the Sinan district's frost, HL, ML, and LL is roughly 2, 75, 23, and 0%, respectively. Frost, HL, ML, and LL agroecologies, respectively, make up about 0, 3, 81, and 16% of the Aneded district. In the Enemay district, the proportions of frost, HL, ML, and LL agroecologies are around 0, 7, 88, and 5%, respectively. Frost, HL, ML, and LL agroecologies make up about 0, 0, 46, and 54%, respectively, of the Basoliben district. Frost, HL, ML, and LL agroecologies, respectively, make up approximately 0, 0, 47, and 53% of the Debre Elias district.

### 2.2. Sampling Techniques and Sample Size

The East Gojjam zone was divided into three strata for the study based on agroecological differences (HL, ML, and LL). Two districts, namely, Debre Elias and Basoliben in LL agroecology, Aneded and Enemay in ML agroecology, and Sinan and Machakel in HL agroecology, were specifically chosen from each agroecology. Then, two peasant associations from each of the six districts were purposely chosen, representing the appropriate agroecologies. As a result, 12 peasant associations were ultimately chosen from among the six districts. According to the Cochran formula, the study sample size from each peasant association was as follows: *n*=(*N*/1+*N*(*e*2)) where *n* = sample size, *N* = the total number of households in the study area, *e* = the maximum variability, and 1 = the probability that the event is occurring. Therefore, a total of 120 households from the six respondents were included in the study's sample size in order to estimate the DM yield of crop residues.

### 2.3. Techniques of Data Collection

Data were mostly collected during the study through questionnaires and field observations. The types of crops and yields of crops were collected by the individual interview questionnaires. In addition, information obtained from group discussions and individual interviews was confirmed through key informant interviews and consultations with district and peasant association livestock experts.

### 2.4. Estimation of Yields of Crop Residues

Using questionnaires in visiting households, crop yields were surveyed. As a result, the crop residue ratio recommended by [4] was used to estimate the crop residues that were now available. To estimate the yield of crop residues from the grain yield, multiplying factors of 2.5 and 2.0 were used for sorghum and maize stover, 1.5 for small cereals, 1.2 for pulse and oil crops, and 0.3 for potatoes. Around 10% of crop residues were thought to have been wasted, either during utilization, used for other reasons, or both [5].

### 2.5. Nutritional Analysis of Feed Samples

To evaluate the nutritional makeup of crop residues, samples of the major crop residues were collected from the districts, and three composite samples of the major crop residues were formed from the three agroecologies. Feed samples were weighed using an electrically sensitive balance and put in a paper bag with the appropriate label. The samples were ground in a Willey mill to pass through a 1 mm sieve screen after drying in an oven at 65°C for 72 hours. The ground samples were transported to the Debre Birhan Agricultural Center for nutritional composition analysis. Feed samples were analyzed for crude protein (CP) using the method [17], and DM, ash, neutral detergent fiber (NDF), acid detergent fiber (ADF), and acid detergent lignin (ADL) were analyzed using the method [18].

### 2.6. Data Analysis

Data were analyzed using SPSS (version 25). The following statistical model was utilized: *Y*_*ij*_=*μ*+*A*_*i*_+*E*_*ij*_, where *Y*_*ij*_ = means for the response variable; *μ* = overall mean; *a* = effects of agroecology; and *e* = error term. The least significant difference was used to test for significant differences between mean comparisons.

## 3. Results and Discussion

### 3.1. Yields of Crops per Household

**Figure 3:**
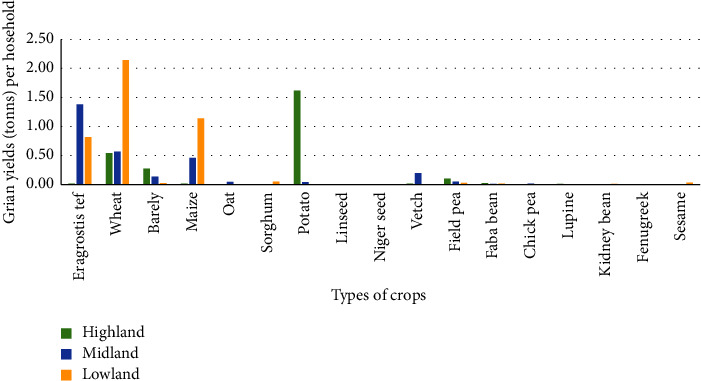
Grain yield (*t*) per household for different crops in different agroecologies.

*Eragrostis tef*, wheat, barley, oats, maize, sorghum, potato, linseed, Niger seed, vetch, field pea, faba bean, chickpea, lupine, kidney bean, and fenugreek were the types of crops grown in the East Gojjam zone (Figure 3). The yield of crops per household varies significantly (*P*  <  0.05) across agroecologies (Table 1). HL agroecology had significantly (*P*  <  0.05) more barley grains per household than ML and LL agroecologies. This occurred as a result of the adaptation of barley to cooler climatic conditions [19], which led to higher barley yields and might be due to the allocation of more land for barley cultivation. However, LL agroecologies had significantly (*P*  <  0.05) greater grain yields of wheat and maize per household than HL and ML agroecologies. This was attributed to the availability of more land for the cultivation of wheat and maize, as well as the environmental adaptability of both maize and wheat in hotter climatic conditions [20]. In contrast, the average yield of *Eragrostis tef* owned by each household in ML agroecology was significantly (*P*  <  0.05) greater than that owned by each household in HL and LL agroecologies, which was similar to the findings reported in [5]. This was related to the adaptation to its environment and the allocation of more land for teff cultivation.

In comparison to cereal crops, the contribution of grain yield from pulse and oil crops was low (4.9% and 0.1%, respectively) and was lower than 12.9% and 5.9% recorded by the CSA in 2021. This might be attributed to environmental conditions such as climate, disease, and soil fertility, as well as the small area of land allocated for pulse and oil seed crops [21]. However, in the ML, as compared to HL and LL agroecologies, the contribution of the yield of the vetch crop was significantly greater (*P*  < 0.05) (Table 2). In general, LL and ML agroecologies had higher crop production per household than HL agroecology. This could be a result of the conversion of grazing land to cropland and the density of the human population.

### 3.2. Production of Crop Residue

**Figure 4:**
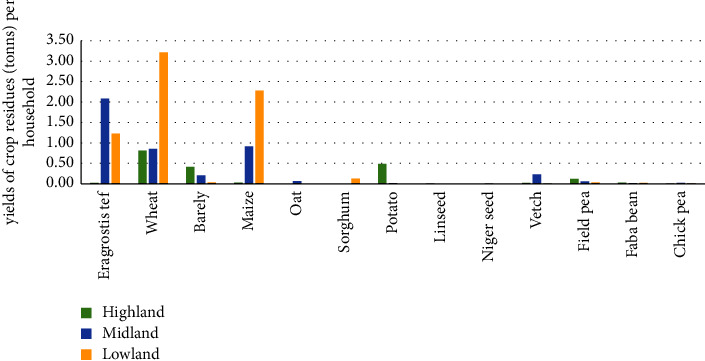
Crop residue yield (*t*) per household in the three agroecologies.

The main crop residues in the current study areas were wheat, *Eragrostis tef*, barley, and maize, as presented in Figure 4. In the study areas, each household produced 4.5 tons (*t*) of DM feed each year from crop residues (Table 2). This quantity was less than the 10.3 tons of DM recorded by [22] for the Horro and Guduru. However, the average amount of DM produced per household from crop residues in this study (4.5 tons) was close to 4.2 tons in the Chire district, southern Ethiopia [22]. There were significant differences (*p*  <  0.05) in the DM yield of crop residues per household across agroecologies. As compared to ML and LL agroecologies, the average amount of crop residues produced per household was lower in HL agroecology, which might be a result of the HL agroecology's inability to produce a variety of crops, the size of cropland, the conversion of forest and pasture land to crop land, and the number of people living in ML and LL agroecologies. This was in contrast to the findings in [22], which reported that the overall average DM yield produced annually from crop residues per household was higher in the HL agroecology than in the of ML agroecology.

In line with the findings in [16], the mean yield of barley and potato residues owned by each household in HL agroecology was significantly higher (*P* < 0.05) than in the ML and LL agroecologies. This might be brought on by the fact that more barley-harvested straws were produced as a result of barley receiving more land, barley being able to adapt to the cold environment, and barley being a strong choice for agroecology [19]. Conversely, the average yield of *Eragrostis tef* straw owned by each household in ML agroecology was significantly higher (*P*  <  0.05) than that owned by each household in the HL and LL agroecologies, which was consistent with the finding from [22]. The reason for this could be that more straw from *Eragrostis tef* was correlated with environmental adaptations of *Eragrostis tef* in ML agroecology and the allocation of more land to *Eragrostis tef* cultivation. However, the average yield of wheat straw and maize stover owned by each household in LL agroecology was significantly higher (*P*  <  0.05) than that owned by each household in the HL and ML agroecologies. This was as a result of more wheat and maize residues being produced as a result of more land being allocated for the cultivation of these two crops and their environmental adaptation to heat [20].

### 3.3. Nutritional Composition of Crop Residues

The nutritional composition of different crop residues is shown in Table 3. Crop residues have CP content ranging from 2.6% (wheat straw in the HL) to 6.2% (faba bean haulms in HL agroecology). The faba bean haulms had the largest amount of CP, followed by vetch, barley, *Eragrostis tef*, wheat straws, and maize stover. However, in all agroecologies, there was a significant difference in the CP content of crop residues (*P* <  0.05). Similar to the findings in [3], the CP level of barley and *Eragrostis tef* straws showed a significant difference (*P* <  0.05) between agroecologies (Table 3). This study showed that the CP levels of *Eragrostis tef*, barley, and wheat straws were higher than those reported in [3], which reported CP levels of *Eragrostis tef*, barley, and wheat straws of 3.6%, 4.9%, and 2.7%, respectively. In this investigation, maize stover had a CP level of 2.9%, which was greater than the figure of 2.7% given in [23]. However, the CP content of maize stover in the present study was less than 3.6% [24] and 3.8% [3]. These variations could be a result of crop variety, fertiliser rate, soil status, variation in different parts of the plant [25], location, temperature, harvest stage of crops, length of storage, and storage conditions [26]. Another author [27] claimed that the lower CP concentration of crop residues may be connected to the longer time needed for the crop to reach physiological maturity, which results in CP dilution and increased lignification.

Since the CP content is less than 7.2% DM, all straws and stovers from different crops in the study areas cannot meet even the maintenance requirements of ruminants [28]. For growth and lactation, ruminants require at least 0.15 kg of CP per kilogram of DM, and for rumen function, they require 0.07 kg of CP per kilogram of DM [29, 30]. As a result, and in line with the findings of [22], the total amount of crop residues in the study area was below what was required for maintenance, rumen function, and livestock production. This indicates that a reasonable livestock production in the studied areas requires the supplementation of protein-source feed and the enhancement of the nutritional value of crop residues by physical, biological, and chemical processes.

According to the findings, the ash content of *Eragrostis tef* and wheat straws was significantly varied (*P*  <  0.05) across agroecologies (Table 3), in agreement with the findings of [3, 31]. *Eragrostis tef* straw in the current study had a lower ash level (6.3%) than *Eragrostis tef* straw in the Tanqua-Abergelle areas in central Tigray, as reported in [32], which had higher ash content (7.15%). However, the ash content of the maize stover in the present study (6.5%) was greater than the ash content (4.4%) of the same grain in the Tanqua-Abergelle areas of central Tigray, as reported in [32]. This fluctuation may be caused by crop management techniques, soil status, temperature, crop harvesting, and crop variety variation.

In the current study, barley straw had the highest level of NDF (76.6%), while faba bean haulms had the lowest NDF content (67.2%). However, there was no significant variation in the NDF content of crop residues across agroecologies, with the exception of wheat straws, which showed a significant difference (*P*  <  0.05) between ML and LL agroecologies, which is consistent with the results of [33] in the south-west Shoa zone, central Ethiopia. The current study reports a higher NDF content for crop residues than the range of 63.0–75.1% reported by [3]. The higher NDF level of crop residues is mainly due to the longer time needed for maturity in the region, which provided a chance for fiber accumulation in plant tissues. In the current investigation, cereal crop residues had higher NDF content than pulse haulms, which was consistent with the findings in [32]. According to [34], roughage feeds with NDF contents of less than 45, 45–65, and more than 65% are classified as high, medium, and low quality, respectively. Since all crop residues in the current study had NDF contents greater than 65%, they are classified as low-quality feed, which indicates that they could impact feed intake and affect their ability to produce and function [15] unless crop residues are treated using physical, biological, and chemical processes.

As shown in Table 3, there were differences in the ADF contents of crop residues across agroecologies. In the current study, barley straw had the highest ADF contents (63.0%), and maize stover had the lowest ADF contents (51.6%), which was in line with results from [33] in the south-west Shoa zone. However, compared to crop residues in the Tanqua-Abergelle district, the majority of crop residues in the present study had the highest ADF contents [32]. This could have influenced the leaf-to-stem ratio, which was thought to be a significant contributor to the nutritional variation in crop residues [35].

The overall ADL concentration of crop residues varies based on the type of crop and across agroecologies. The crop residues in the study areas had lignin contents between 7.8 and 11.3%, which were lower than 5 to 20% documented in [32]. However, the lignin content of the majority of the crop residues in the current study was greater than the lignin content of the majority of crop residues in the Tanqua-Abergelle region, which ranged from 5.6 to 6.6% [32]. According to [29], lignin is the single most important factor in limiting feed intake, the rate of organic matter fermentation, the number of microbial cells produced per unit of fermented organic matter, and the proportion of propionate to acetate in the products of fermentation. The percentage of fiber that is digested may be less than 60% in feed that contains 100 g/kg of lignin [35]. As a result, the lignin content of the crop residues in the study areas was close to 100 g/kg, which restricts the amount of DM that livestock may consume and livestock production. Therefore, it is essential to provide livestock with protein feed and treat crop residues with urea to boost the feeding value of low-quality crop residues, because crop residues can be treated with urea or efficient microorganisms to produce a product with higher nutritional value, especially when the untreated product has a very low nutritional value [36, 37].

## 4. Conclusions

Crop residues are a popular source of animal feed in many agricultural areas, and the most accessible forms of crop residues in the East Gojjam zone are wheat, *Eragrostis tef*, barley, and maize. These residues were all found to have low quantities of CP, which is an important nutrition for animals. They also have significant quantities of NDF, which might influence feed intake and cattle output. As a result, it is critical to improve crop residue quality through mechanical, biological, and chemical means in order to maximize feed intake and, eventually, livestock output.

## Figures and Tables

**Table 1:** Grain yield (*t*) of different crops at different agroecologies.

Crop type	Scientific name	Agroecologies				*P* value
		Highland	Midland	Lowland	Mean	
Teff	*Eragrostis tef*	0.15 ± 0.56^a^	13.83 ± 0.35^b^	8.15 ± 0.13^c^	7.53 ± 0.4	0.000
Wheat	*Triticum aestivum* L.	5.39 ± 0.42^a^	5.68 ± 0.83^b^	21.44 ± 0.9^b^	9.89 ± 0.71	0.003
Barley	*Hordeum vulgare* L.	2.77 ± 0.44^a^	1.36 ± 0.08^b^	0.22 ± 0.63^c^	1.45 ± 0.11	0.030
Maize	*Zea Mays* L.	0.15 ± 0.77^a^	4.58 ± 0.74^b^	11.39 ± 0.1^c^	4.9 ± 0.0.24	0.000
Oats	*Avena sativa*	0.00 ± 0.00^a^	0.44 ± 0.61^a^	0.00 ± 0.00^a^	0.17 ± 0.06	0.113
Sorghum	*Sorghum bicolor*	0.00 ± 0.00^a^	0.00 ± 0.00^a^	0.52 ± 1.38^b^	0.14 ± 0.75	0.010
Potato	*Solanum tuberosum* L.	16.17 ± 0.8^a^	0.42 ± 0.04^b^	0.00 ± 0.00^c^	5.93 ± 0.21	0.000
Linseed	*Linum usitatissimum*	0.04 ± 0.12^a^	0.00 ± 0.00^a^	0.00 ± 0.00^a^	0.01 ± 0.07	0.069
Niger seed	*Guizotia abyssinica*	0.00 ± 0.00^a^	0.05 ± 0.20^a^	0.00 ± 0.00^a^	0.02 ± 0.12	0.199
Vetch	*Vetch species*	0.15 ± 0.87^a^	1.95 ± 0.28^b^	0.04 ± 0.20^a^	0.80 ± 0.74	0.000
Field pea	Pisum sativum L.	1.00 ± 0.02^a^	0.50 ± 0.03^a^	0.30 ± 0.06^a^	0.60 ± 0.74	0.445
Faba bean	*Vicia faba* L.	0.23 ± 0.50^a^	0.13 ± 0.52^a^	0.17 ± 0.54^a^	0.17 ± 0.0.5	0.721
Chickpea	*Cicer arietinum* L.	0.06 ± 0.34^a^	0.17 ± 0.64^a^	0.10 ± 0.34^a^	0.11 ± 0.47	0.650
Lupine	*Lupine species*	0.12 ± 0.48^a^	0.00 ± 0.00^b^	0.00 ± 0.00^a^	0.04 ± 0.0.28	0.150
Kidney bean	*Phaseolus vulgaris* L	0.00 ± 0.00^a^	0.00 ± 0.00^a^	0.12 ± 0.43^a^	0.032 ± 0.0.22	0.089
Fenugreek	*Trigonella foenum-graecum* L.	0.00 ± 0.00^a^	0.02 ± 0.10^a^	0.00 ± 0.00^a^	0.006 ± 0.06	0.445
Sesame	*Sesamum indicum* L.	0.00 ± 0.00^a^	0.00 ± 0.00^a^	0.31 ± 0.12^a^	0.08 ± 0.59	0.079
Total						0.018

The means in the same row that have different superscript letters differ considerably (*P*  <  0.05).

**Table 2:** Average crop residue yield (in tons) per household in the study areas.

Crop types	Altitudes				*P* value
	Highland	Midland	Lowland	Average	
Teff	0.02 ± 0.50^a^	2.08 ± 0.42^b^	1.22 ± 0.12^c^	1.11	<0.001
Wheat	0.81 ± 0.52^a^	0.85 ± 0.89^a^	3.22 ± 0.42^b^	1.63	<0.001
Barley	0.42 ± 0.10^a^	0.02 ± 0.23^b^	0.03 ± 0.61^c^	0.22	0.020
Maize	0.03 ± 0.17^a^	0.92 ± 0.32^b^	2.28 ± 0.22^c^	1.07	<0.001
Oats	0.00 ± 0.00^a^	0.07 ± 0.90^a^	0.00 ± 0.00^a^	0.02	0.100
Sorghum	0.00 ± 0.00^a^	0.00 ± 0.00^a^	0.13 ± 0.63^b^	0.04	0.045
Potato	0.49 ± 0.34^a^	0.01 ± 0.50^b^	0.00 ± 0.00^b^	0.17	0.034
Linseed	0.01 ± 0.02^a^	0.00 ± 0.00^a^	0.00 ± 0.00^a^	0.00	0.081
Niger seed	0.00 ± 0.00^a^	0.01 ± 0.40^a^	0.00 ± 0.00^a^	0.00	0.232
Vetch	0.02 ± 0.28^a^	0.23 ± 0.33^b^	0.01 ± 0.48^a^	0.09	0.030
Field pea	0.12 ± 0.69^a^	0.06 ± 0.45^a^	0.04 ± 0.46^a^	0.07	0.445
Faba bean	0.03 ± 0.56^a^	0.02 ± 0.20^a^	0.02 ± 0.30^a^	0.02	0.249
Chick pea	0.01 ± 0.35^a^	0.02 ± 0.15^a^	0.01 ± 0.31^a^	0.01	0.904
Total	**1.94**	**4.47**	**6.95**	**4.45**	

The means in the same row that have different superscript letters differ considerably (*P*  <  0.05).

**Table 3:** Nutritional composition of different crop residues in the three agroecologies.

Crop residues	Agroecologies	DM %	ASH %	OM %	CP %	NDF %	ADF %	ADL %
Teff straw	Highland	92.00 ± 0.72^a^	4.35 ± 0.49^b^	87.65 ± 0.21^a^	3.08 ± 0.77^b^	68.22 ± 0.27^a^	53.76 ± 0.42^a^	9.62 ± 0.04^a^
	Midland	92.33 ± 0.76^a^	7.57 ± 0.76^a^	84.76 ± 0.43^a^	3.51 ± 0.77^b^	70.02 ± 0.16^a^	54.48 ± 0.42^a^	8.91 ± 0.04^a^
	Lowland	92.51 ± 0.56^a^	5.41 ± 0.55^b^	87.10 ± 0.97^a^	4.25 ± 0.82^a^	71.40 ± 0.59^a^	56.67 ± 0.75^a^	9.77 ± 0.11^a^
	Average	92.33	6.31	86.02	3.69	70.18	55.10	9.31

Barley straw	Highland	92.0 ± 1.00^a^	4.9 ± 1.59^a^	87.1 ± 0.59^a^	6.04 ± 0.14^a^	74.30 ± 0.31^a^	60.0 ± 0.972^a^	10.0 ± 0.97^a^
	Midland	93.0 ± 1.41^a^	4.3 ± 1.84^b^	88.7 ± 0.25^a^	4.50 ± 0.20^b^	76.64 ± 0.34^a^	62.1 ± 0.9.72^a^	11.3 ± 0.97^a^
	Average	92.33	4.70	87.60	5.53	75.06	63.03	10.4

Wheat straw	Highland	92.00 ± 1.36^a^	3.80 ± 0.87^b^	88.20 ± 0.61^a^	3.32 ± 0.42^a^	73.55 ± 0.17^a^	59.14 ± 0.84^a^	10.01 ± 0.91^a^
	Midland	93.00 ± 1.36^a^	5.38 ± 0.87^a^	87.62 ± 0.50^a^	2.59 ± 0.34^a^	75.37 ± 0.40^a^	60.22 ± 0.30^a^	9.91 ± 0.62^a^
	Lowland	92.50 ± 1.11^a^	4.86 ± 0.71^a^	87.64 ± 0.50^a^	3.45 ± 0.42^a^	68.95 ± 0.16^b^	53.76 ± 0.30^b^	9.17 ± 0.76^a^
	Average	92.40	4.54	87.82	3.23	72.01	57.20	9.65

Maize stover	Lowland	92.00 ± 0.23	6.52 ± 0.87	85.48 ± 0.59	2.85 ± 0.17	67.37 ± 0.31 ^a^	51.61 ± 0.30	7.8 ± 0.85
Faba bean	Highland	92.00 ± 0.32	5.49 ± 0.23	85.51 ± 0.12	6.19 ± 0.39	67.22 ± 0.64 ^a^	55.91 ± 0.76	9.62 ± 0.34
Vetch straw	Midland	92.33 ± 0.57	4.49 ± 0.32	87.84 ± 0.64	5.30 ± 0.69	69.30 ± 0.06	54.48 ± 0.89	9.31 ± 0.72
	Overall	**92.33**	**5.19**	**87.05**	**4.10**	**71.12**	**56.56**	**9.58**

Means within a single column with various superscript letters differ significantly (*P* < 0.05) across agroecologies; DM = dry matter; OM = organic matter; CP = crude protein; NDF = neutral detergent fiber; ADF = acid detergent fiber; ADL = acid detergent lignin. The significance of the bolded values in Table 3 is provided to help readers understand the overall nutritional composition of crop residues.

## Data Availability

The data supporting this work are available from the author upon request.
